# Biological Significance of Calbindin-D_9k_ within Duodenal Epithelium

**DOI:** 10.3390/ijms141223330

**Published:** 2013-11-26

**Authors:** Eui-Ju Hong, Eui-Bae Jeung

**Affiliations:** Laboratory of Veterinary Biochemistry and Molecular Biology, College of Veterinary Medicine, Chungbuk National University, Cheongju, Chungbuk 361-763, Korea; E-Mail: ejhong@chungbuk.ac.kr

**Keywords:** calbindin, transcellular pathway, vitamin D receptor, duodenum, calcium absorption, knockout mice

## Abstract

Calbindin-D_9k_ (CaBP-9k) binds calcium with high affinity and regulates the distribution of free calcium in the cytoplasm. The expression of CaBP-9k is detected primarily in intestine that is vitamin D target tissue, and accumulates in the enterocytes of the duodenal villi. These enterocytes are the clearest example of vitamin D responsive cells, and the presence of CaBP-9k within them accentuates calcium absorption mediated by active transcellular calcium transport. It has been well established that the expression of CaBP-9k is mediated with vitamin D response element on its promoter and it regulates the amount of intracellular calcium in order to prevent cell death from reaching the toxicity of free calcium. There is now little doubt that glucocorticoid also decreases CaBP-9k expression in duodenal epithelial cells. In addition, it was reported that the level of *CaBP-9k* gene in enterocytes is increased in pregnancy when the plasma estradiol concentration is generally associated with a concomitant increase. Although calcium homeostasis was not disturbed in mice lacking the *CaBP-9k* gene, we found that CaBP-9k has a buffering role of free calcium in the cytosolic environment beyond that of calcium transfer. To expand our knowledge of the biological functions of CaBP-9k, our research has focused on defining the biological significance of intracellular CaBP-9k. Our findings suggest that the *CaBP-9k* gene is involved in compensatory induction of other calcium transporter genes in duodenal epithelial cells. This article summarizes the findings from recent studies on the expression and the functions of CaBP-9k in the small intestine.

## 1. Introduction

**Figure 1.**
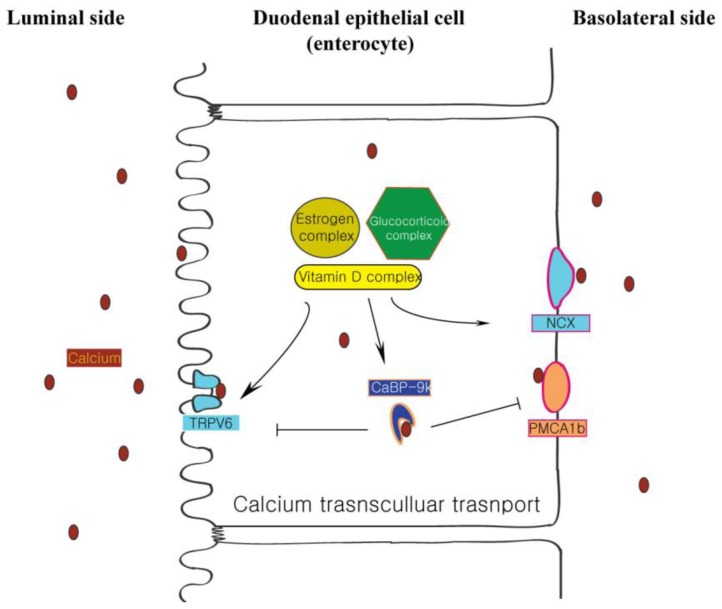
CaBP-9k of transcellular calcium transport in duodenal enterocyte. In the transportation, calcium binds to membrane bound TRPV6, which influxes from the extracellular environment. The calcium translocates into the cytoplasm and binds to calcium binding protein CaBP-9k. The calcium-bound CaBP-9k may also move to the basolateral side and transfer its bound calcium to PMCA1b or NCX channel protein, leading to uptake into serum.

Calcium homeostasis refers to the regulation of the concentration of calcium ions in the body, and impairment of this mechanism contributes to their underlying pathologies, such as hypercalcemia or hypocalcemia. When calcium is absorbed in the intestine along either transcellular or paracellular routes, the transcellular transport of calcium ion occurred mainly in the upper intestine including the duodenum and upper jejunum. As a component of active transcelluar trasnport process, CaBP-9k (S100G in human) was originally described as a vitamin D-dependent calcium-binding protein in the intestine [1], and it binds intracellular calcium with high affinity in the cytoplasm [2]. In addition, there is evidence that CaBP-9k regulates the level of intracellular free calcium in order to prevent this mineral from reaching toxic levels [3]. Traditionally, it was thought that intracellular binding proteins transfer cytosolic calcium from the apical membrane to the basolateral membrane via trans-cellular transport of calcium [4], while calcium entry is facilitated by apical transient receptor potential vanilloid 6 (TRPV6), and calcium export is promoted by plasma membrane Ca^2+^-ATPase 1b (PMCA1b) as shown in Figure 1. PMCA1b facilitates the excretion of calcium ions using adenosine triphosphate (ATP) hydrolysis [5]. Like PMCA1b, NCX1 exchanges outer sodium ions for inner calcium ions [6,7]. In order to understand the influence of CaBP-9k related to other calcium related proteins, we focused our attention on the expression and function of CaBP-9k within duodenal epithelial cells.

**Figure 2.**
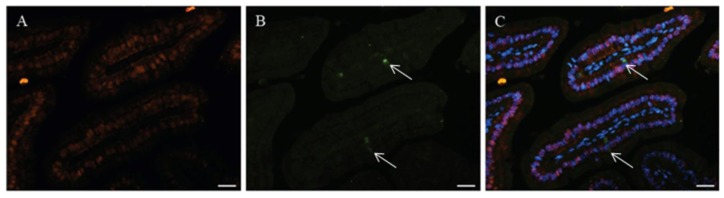

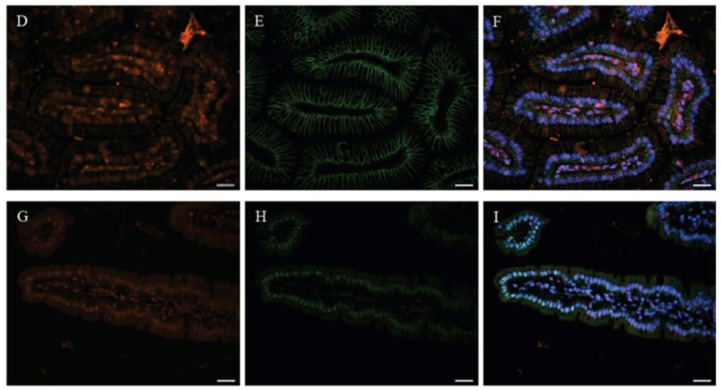
Immuno-histochemical analyses in duodenal villi of mice. **A** to **F**, immunoreactive CaBP-9k appears in the cytoplasm of many populations of epithelial cells within the duodenal villi (**A** and **D**, **red**) at 100× magnification by immunofluorescence detection. To identify the co-localization between CaBP-9k and other proteins, the specific antibodies for chromogranin A (**B**, **green**) and E-cadhedrin (**E**, **green**) were co-incubated with anti-mouse CaBP-9k antibodies using corresponding Alexa-Fluor conjugated secondary antibodies. DAPI (**blue**) was used for nuclei staining. Based on DAPI signals, co-localization of CaBP-9k and each protein is evident by superimposition (**C** and **F**) of the green and red signals, respectively. HNF-4α (nuclear protein marker, **G**, **Red**) used in an attempt to identify the GR (**H**, **green**) expression in duodenal enterocytes, both images were merged and observed as blue (**I**). Scale bar = 20 μm.

The duodenum is the first part of the small intestine in which digested nutrients are absorbed by the epithelial layer. Although the jejunum and ileum are also other sites of dietary calcium absorption via paracellular absorption [8,9], the presence of calcium-related proteins in duodenal epithelial cells also promotes uptake of calcium across the epithelium and its transport into the blood stream via transcellular absorption. We have previously shown that in the porcine intestine CaBP-9k is highly expressed in the duodenum. CaBP-9k expression decreases gradually aborally to undetectable in the distal ileum [10]. In the intestinal epithelium, three differentiated types of cells along the villus are organized with enterocytes, goblet cells, and enteroendocrine cells [11]. As intestinal absorptive cells, enterocytes are terminally differentiated cells comprising the majority of the intestinal epithelium. To further define which epithelial cell types within the duodenum express CaBP-9k, we examined in our unpublished study the duodenum of mice by double-label immuno-histochemical studies with anti-mouse CaBP-9k antibody and antibody directed against chromogranin A (enteroendocrine cell marker) or E-cadhedrin (epithelial cell maker). As previously reported for enterocyte [12], immune-reactive CaBP-9k presents in the majority of the intestinal epithelium as shown in Figure 2, and this suggest that mouse CaBP-9k in the duodenum might be confined to enterocytes, the major population of duodenal epithelial cells, because other cell types in the epithelium are present in 2%–3% population of epithelial cells in the villus.

## 2. Vitamin D-Dependent CaBP-9k Expression in the Small Intestine

It is generally accepted that vitamin D_3_ plays a key role in calcium uptake, and there is evidence that vitamin D_3_ is activated by 25-hydroxylation and 1α-hydroxylation in the liver and kidney, respectively. Metabolized calcitriol (1,25-dihydroxyvitamin D_3_) enters enterocytes by passive diffusion and interacts with the nuclear vitamin D receptor (VDR), which heterodimerizes with the retinoid X receptor [13]. Knockout mice lacking VDR or 1α-hydroxylase have previously been used for evaluation of the effects of vitamin D on expression of the calcium transport gene and transcellular calcium transport [14,15]. Because calcitriol is essential for intestinal calcium absorption [16], this calciotropic hormone facilitates maintenance of calcium homeostasis via the VDR by acting on the vitamin D response element (VDRE) in target genes, such as *CaBP-9k* [17] and *TRPV6* [18]. More importantly, VDR is expressed in epithelial cells involved in calcium absorption. Using knockout mouse models, deficiencies in plasma calcitriol or intracellular VDR expression were found to show an association with the development of rickets and hypocalcemia [14,19]. In 1α-hydroxylase knockout mice, reduced plasma calcium concentrations are restored to normal levels by exogenous calcitriol, suggesting that this hormone induces calcium uptake mediated by expression of calcium transporters [20]. Therefore, it was concluded that expression of CaBP-9k mRNA and protein is induced by calcitriol in the intestine due to the presence of a VDRE in the *CaBP-9k* gene promoter [21]. Expression of the CaBP-9k mRNA in the intestine is substantially reduced in mice lacking 1α-hydroxylase, a metabolic enzyme for the production of calcitriol [14]. Regardless of the presence of calcitriol, intestinal CaBP-9k mRNA and protein was reduced in VDR-null mice [22], and this observation suggests that CaBP-9k transcription is mediated by binding of ligand-bound VDR to the VDRE located within the promoter regions. Although vitamin-D-mediated regulation is involved in reproductive tissues [19,23], we confirmed that CaBP-9k mRNA does not respond to vitamin-deficient diet or active vitamin D despite the presence of VDR in the uterus, as described previously [24].

To further determine whether the calcium-binding properties of CaBP-9k in gastrointestinal tissues are responsible for the enhanced VDR expression, we explored the link between CaBP-9k and VDR expression in human intestine. Following the increase of CaBP-9k mRNA and decrease of blood calcium revealed in an age-dependent manner, our study shows that the link between age and VDR mRNA expression in the human duodenum does not appear to correlate [25]. While an earlier study reported that the levels of active vitamin D (calcitriol) are reduced in an age-dependent manner [26], we failed to address the link between CaBP-9k levels and calcium absorption because calcium absorption declines even when the level of CaBP-9k mRNA remains high in the duodenal epithelium [25]. These results imply that CaBP-9k may not be a critical factor for duodenal calcium absorption, and the latter was confirmed by our previous study using a CaBP-9k knockout mouse model [27]. Although the calcium absorption does not correspond with CaBP-9k expression in the duodenum, it is still considered that CaBP-9k has a role as a transport protein because it increases the total amount of intracellular calcium [9].

## 3. Other Physiological Factors that Affect CaBP-9k Expression

Glucocorticoids have been reported to inhibit calcium absorption by reducing active transport and brush border uptake [28,29]. Although renal CaBP-9k levels were significantly decreased in adrenalectomized mice from which endogenous cortisol had been removed by a surgical method, we did not observe any difference in expression of intestinal CaBP-9k mRNA or protein, compared with sham-operated animals. It is widely accepted that glucocorticoids reduce intestinal calcium absorption by inhibition of active calcium transcellular transport and brush border vehicle uptake [29,30]. In addition, cortisol reduced the production of intestinal calcium binding protein in chicks [31]. Previous studies have reported that the glucocorticoid receptor (GR) is highly expressed in epithelial cells of the duodenum, and glucocorticoid appears to control differentiation and mineral absorption of the epithelial cell [32,33]. We observed that accumulation of CaBP-9k occurs within the cytoplasm of the same duodenal epithelium where we detected GR expression in the nuclei as shown in Figure 2. After treatment with dexamethasone, used as a GR agonist, the level of CaBP-9k expression was substantially decreased in duodenal enterocyte [34]. We also demonstrated that the reduced expression of duodenal CaBP-9k via dexamethasone administration is reversed by treatment with a GR antagonist [34], and our data all suggest that the ligand-bound GR decreased CaBP-9k expression in duodenal epithelial cells. In addition to the intestine, we also observed that the reduction of renal CaBP-9k expression in adrenalectomized mice was restored by administration of dexamethasone [34]. Renal CaBP-9k proteins expressed in the distal convoluted tubule have been suspected of active calcium reabsorption [34], like a function of CaBP-28K which has been implicated in active calcium absorption in the kidney [35].

We have observed that CaBP-9k expression is induced in the uterus and pituitary glands by endogenous/exogenous estrogens [36–38]. Although the expression and regulation of CaBP-9k in these tissues have been well described, the regulation of CaBP-9k expression remains obscure in the duodenum. At least, the promoter region of *CaBP-9k* gene contains a putative estrogen receptor-binding site located in the first intron [39]. In an earlier study, the regulation of CaBP-9k expression resulted in response in the rat uterus by estrogen, but not in the intestine [40]. Nevertheless, after menopause estrogen deficiency is associated with increased renal calcium loss [41]. Interestingly, there is considerable evidence that estrogen has a physiological role in regulation of intestinal calcium absorption. According to one report, estrogen might promote calcium absorption by increasing the expression of apical channels in duodenal epithelial cells [12]. In our recent study, the expression of duodenal CaBP-9k mRNA was disrupted by exogenous estrogen or an estrogenic compound during late pregnancy, when endogenous estrogen production shows a significant increase [42]. This effect of exogenous compound may disrupt the activity of the estrogen receptor, which controls an estrogen-regulated CaBP-9k expression.

**Figure 3.**
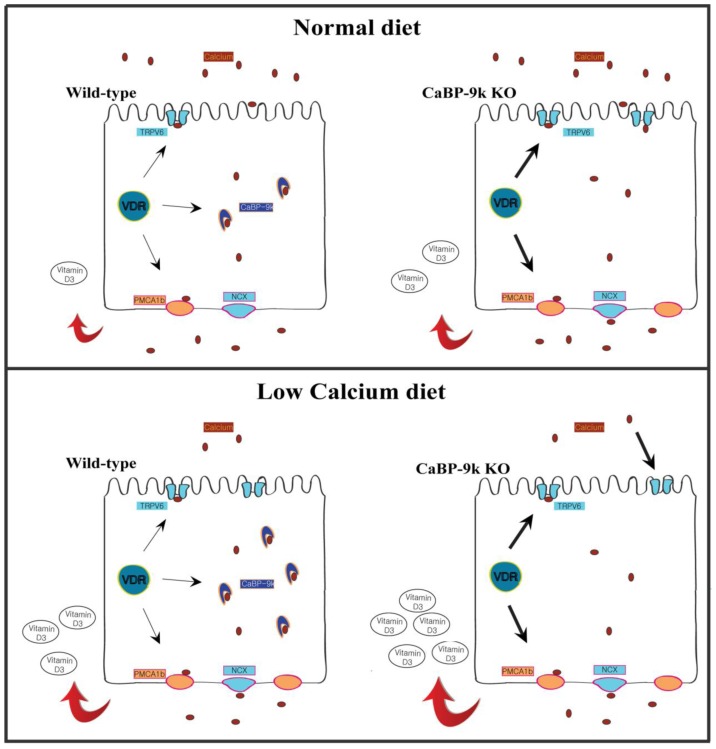
The role of CaBP-9k in the extracellular and intracellular environment of enterocytes. In calcium absorption, the abolishment of intracellular CaBP-9k stimulates compensative increase of vitamin D, and the increased vitamin D induces the expression of apical or basolateral membrane bound channels. In addition, a low calcium diet may also stimulate the expression of membrane bound channels in CaBP-9k knockout mice.

Since the intestinal calcium transporting system was activated in mice fed a low-calcium diet, dietary calcium is also considered to be another regulator of *TRPV6* or *CaBP-9k* gene expression [43]. For example, TRPV6 or CaBP-9k mRNA expression of wild-type mice was higher in a low-calcium diet compared to a normal-calcium diet as shown in Figure 3. In addition, the TRPV6 expression in CaBP-9k-null mice was also higher than that of wild-type mice when the mice were fed a normal diet. CaBP-9k was also reported to regulate calcium influx across the TRPV channel by buffering intracellular calcium [44]. When the animals were fed a low calcium diet, the serum calcitriol level was significantly increased in CaBP-9k null mice compared with the wild-type mice [27]. Intuitively, one might suspect that the absence of *CaBP-9k* gene would accelerate the production of calcitriol in the kidney, because this hormone is generally considered to be the main regulator of TRPV6 expression. It was also reported that the repression of PMCA1b expression was shown by calcium deficiency, but NCX1 expression was stimulated in the duodenum by low calcium diet [27]. Furthermore, duodenal PMCA1b mRNA in VDR-null mice was reduced by a calcium deficiency diet [12]. These reports raise the possibility that dietary calcium can regulate the expression of other membrane-bound calcium channels beyond that of intracellular calcium binding protein as shown in Figure 3.

## 4. The Influence of Intracellular CaBP-9k in Expression of Other Calcium-Associated Proteins

To further define the role of CaBP-9k in calcium transport, we generated CaBP-9k-null mice and evaluated the phenotype of these animals. While calcium homeostasis is disrupted in VDR-null or 1α-hydroxylase-null mice [20,45], CaBP-9k-null mice were phenotypically normal for the birth and survivality for one year [27]. Since abolishment of *CaBP-9k* gene did not affect calcium absorption or blood calcium levels in the mice, we searched for other factors that might be compensated by CaBP-9k expression. In an earlier study, it was observed that the role of CaBP-9k is to stimulate the rate of extrusion of calcium via increase of the calcium pump [46]. During the pre-weaning period, we found that the absence of CaBP-9k enhances the compensatory expression of membrane-bound calcium channels, such as TRPV6 and PMCA1b [27]. It has also been demonstrated that calcitriol may be involved in the expression of duodenal CaBP-9k expression, but not involved in that of PMCA1b expression [47]. Since VDR expression of CaBP-9k-null mice was similar to that of wild-type mice, the compensatory induction of TRPV6 expression in CaBP-9k null mice may be related to the level of active vitamin D in the blood (Figure 3). We have explored this expectation in a series of experiments that lead us to conclude that the absence of CaBP-9k within specific cell types, such as renal epithelial cells and duodenal epithelial cells, accentuates the vitamin D environment in the body. We demonstrate also that TRPV6 mRNA in CaBP-9k null mice is halted when the animals are fed a vitamin D_3_-deficient diet [48]. In addition, the expression of TRPV6 and PMCA1b revealed a compensatory increase in CaBP-9k null mice as shown in Figure 3, and their inductions are reduced by exogenous glucocorticoid, which regulates duodenal VDR transcription in CaBP-9k null mice [49]. While abolishment of *CaBP-9k* gene did not reveal significant affection for calcium absorption or blood calcium levels, the CaBP-9k could control the blood calcium level via compensatory expression of TRPV6 and PMCA1b. It was reported that intestinal calcium absorption in TRPV6-null mice is limited, and these animals develop calcium deficiency despite activity by calcitriol [50]. Conversely, expression of CaBP-9k mRNA in the intestine of TRPV6-null mice is significantly altered compared to the wild-type counterparts [51]. These findings provide evidence that the absence of intracellular calcium-binding proteins might induce compensatory expression of other genes for maintenance of calcium absorption.

## 5. Conclusions

Since identification of CaBP-9k as a vitamin D-dependent calcium-binding protein within the intestine, its role has been examined in various physiological and experimental environments. Until recently, CaBP-9k was thought to facilitate calcium transfer through the cytosol or aid in regulation of free calcium levels by mediation of calcium absorption. However, our recent studies demonstrated that CaBP-9k is not essential for active intestinal calcium absorption. The CaBP-9k-null mouse model fails to explain how the absence of CaBP-9k in duodenal epithelial cells directly restricts calcium uptake. Nevertheless, our results from the CaBP-9k-null mouse support the concept that CaBP-9k may play a role in calcium absorption of the cytosolic environment during active calcium transport, which could act as a potential modulator of other membrane-bound calcium channels. Since the genetic inactivation of *CaBP-9k* gene does not disrupt the calcium homeostasis, this therefore raises the obvious question of whether the generation of a double or triple knockout mouse model could contribute to impair calcium homeostasis.
